# Perinatal and neonatal outcomes for fetoscopic laser ablation for the treatment of twin twin transfusion syndrome at a single center

**DOI:** 10.1038/s41372-022-01568-2

**Published:** 2022-12-07

**Authors:** Diamond Ling, Alexandra Phelps, Tabitha Tate, James Adefisoye, Suwan Mehra, Preetha Prazad

**Affiliations:** 1 Department of Neonatal-Perinatal Medicine, Advocate Children’s Hospital, Park Ridge, IL USA; 2 Department of Obstetrics and Gynecology, Advocate Lutheran General Hospital, Park Ridge, IL USA; 3 Department of Pediatrics, Advocate Children’s Hospital, Park Ridge, IL USA; 4 Department of Graduate Medical Education, Aurora Sinai Medical Center, Milwaukee, WI USA; 5 Department of Maternal Fetal Medicine, Advocate Children’s Hospital, Park Ridge, IL USA

**Keywords:** Outcomes research, Paediatrics, Diseases

## Abstract

**Objective:**

To describe the perinatal and neonatal outcomes of fetal laser ablation (FLA) for the treatment of twin-twin transfusion syndrome (TTTS) in our single center institution.

**Study design:**

Retrospective study of 76 treated pregnant women. Procedural complications, perinatal and neonatal outcomes analyzed. Differences in outcomes between two procedural techniques, selective and Solomon, compared.

**Results:**

FLA occurred at median gestational age (GA) of 20.8 weeks (IQR 18.1–22.9) with low incidence of procedural complications (5.3%). High survival rate with delivery of at least one neonate (96%) [95% CI: 88.9–99.2%]; 73.7% [95% CI: 62.3–83.1%] were twins. Median GA at birth was 33.1 weeks (IQR 28.0–35.0). Neonatal mortality and morbidities were 9.4% and 48.3% of cases respectively, and associated with lower GA. Solomon cases had comparatively higher median GA, and lower incidences of neonatal morbidities.

**Conclusion:**

Our small single center study showed favorable outcomes for using the Solomon technique in the treatment of TTTS.

## Introduction

Twin-Twin Transfusion syndrome (TTTS) is a complication of monochorionic multi-gestational pregnancies, which when left untreated, can have an overall mortality rate of 73-100% [1]. TTTS occurs in the presence of inter-twin vascular anastomoses on the monochorionic placenta, causing an imbalance of blood flow between fetuses and leading to anemia and anhydramnios in one fetus (“Donor”) and polycythemia and polyhydramnios in another (“Recipient”) [1]. These in utero hemodynamic disturbances increases risk of brain injury and congenital heart disease, such as ventricular dysfunction and cardiomyopathy, which may lead to heart failure [1–3]. Other morbidities associated with TTTS include prematurity, intraventricular hemorrhage (IVH), renal failure, hydrops fetalis, polycythemia/ and hyperviscosity syndrome in the recipient fetus and anemia in the donor fetus and long-term neurodevelopmental impairment [2–5]. In utero cardiac manifestations such as ventricular dysfunction and cardiomyopathy, are associated with increased risk of fetal demise and brain injury [3].

The degree of severity of TTTS is divided into Quintero stages with greater morbidity and mortality associated with higher stages [6]. Fetoscopic laser ablation (FLA) of vascular anastomoses disrupts the communication between the fetuses and drastically improves survival rates and neonatal outcomes [1, 5, 7]. FLA is not without its risks; there is increased risk for preterm premature rupture of membranes (PPROM), and consequently, chorioamnionitis, premature birth and low birth weight [2, 8, 9]. Despite improved survival rates, TTTS treated with FLA is still associated with risk for neurological abnormalities, including IVH, cystic periventricular leukomalacia (PVL), porencephaly and ventriculomegaly [10].

Specific FLA techniques include selective ablation of visualized sites of anastomoses and, more recently developed “Solomon technique”, which involves laser coagulation of the entire vascular equator of the placenta [2, 5, 8]. The principle behind the Solomon technique is to eliminate all potential anastomoses, including vessels that might not be visualized, to reduce recurrence of TTTS as well as the development of post laser Twin Anemia Polycythemia Sequence (TAPS) [5, 8, 9, 11]. Previous cohort studies report that the Solomon technique showed a significant reduction in post laser TAPS and improved fetal mortality without apparent increases in perinatal complications, such as PPROM and chorioamnionitis, or neonatal morbidities, such as premature birth, intraventricular hemorrhage (IVH) or cardiac dysfunction [2, 3, 12, 13]. However, a larger randomized control trial did not find a statistically significant difference in fetal survival rates, though the study may not have been sufficiently powered to measure this outcome [14]. In addition, there are concerns whether lasering apparently healthy placental tissue between the anastomoses is justified as it causes increased placental injury [15]. Studies have reported increased incidence of placental abruption and PPROM associated with the Solomon procedure [16–18].

The aim of this study was to describe the perinatal and neonatal outcomes of FLA for the treatment of TTTS in our single center institution; and to evaluate differences in outcomes between selective FLA and the Solomon technique.

## Subjects and methods

**Fig. 1:**
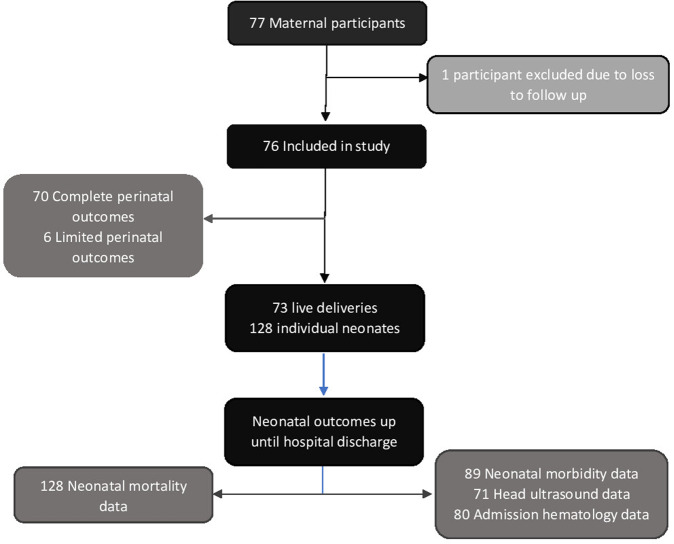
Flow chart diagram of study patients, who underwent FLA 2011–2020. There were 73 live deliveries and 128 individual neonates born. Complete perinatal data was available for 70 maternal patients. Gestational age and neonatal mortality data was available for all neonates, but neonatal morbidity data was limited.

This retrospective observational study investigated outcomes of pregnant women and their neonates after undergoing FLA for the treatment of TTTS at a tertiary level Hospital. 77 maternal patients underwent FLA between October 14, 2011 and December 10, 2020; of these, 1 participant was excluded from the study due to transfer of care and subsequent loss to follow up (Fig. 1). This study was approved by the local Institutional Review Board.

Maternal patients followed by or referred to our center for suspected TTTS underwent a standard evaluation, diagnosed, and staged according to Quintero staging system [1, 6]. Up until February 13, 2016, all 17 maternal patients underwent selective FLA. From May 4, 2016 until December 10, 2020, Solomon technique was used in 36 patients and selective FLA was used in 23 patients. Following FLA, maternal patients either continued routine monitoring at our center or returned to their previous providers for obstetric management and delivery. Electronic medical record and charts were retrospectively reviewed for maternal patients, and their neonates up until initial hospital discharge. Data from maternal patients and their neonates born at an outside institution were limited to records available in EMR from prior transfer of records and patient reports maintained in official records by Maternal-Fetal Medicine team (Fig. 1).

### Data collection

Demographic data of maternal subjects recorded were age, BMI, Quintero staging of TTTS or diagnosis of TAPs, pertinent medical history of pre-eclampsia, gestational diabetes, cervical insufficiency, preterm labor, drug use, mental health diagnoses, advanced maternal age (AMA) status and chronic health conditions. Procedural data collected included gestational age (GA) at time of FLA, incidence of post-procedural TAPs, TTTS recurrence, and in utero fetal demise (IUFD). In the event of IUFD, fetuses were identified by donor or recipient status, latency time from FLA to IUFD and GA at time of IUFD were also recorded. Modes of delivery and perinatal complications commonly associated with TTTS and FLA were included: incidence of placental abruption, chorioamnionitis and PPROM, latency from FLA to PPROM, and GA at time of PPROM [16–18].

Demographic data of neonates included sex, donor-recipient identity, birth weight (BW), GA at birth, presence of twin discordance (defined as weight discrepancy >20%). Incidence of neonatal mortality, corrected GA (cGA) at time of death, and causes of death when known were recorded. Incidence of neonates affected by morbidities associated with complications of TTTS and FLA were recorded [1–3, 12, 13]. These morbidities included early and late onset sepsis, IVH, presence of anemia or polycythemia immediately following delivery with need for blood transfusions, cardiac dysfunction defined as presence of clinically significant patent ductus arteriosus (PDA), hypotension, arrhythmia, congenital heart disease and/or pulmonary hypertension, renal dysfunction defined as presence of acute kidney injury (AKI), renal failure, and congenital anomalies.

### Procedural and perinatal outcomes

The procedural data of interest were GA at time of FLA and the incidence of complications: post-procedural TAPS, TTTS recurrence, and IUFD. Perinatal outcomes examined include incidence of PPROM, placental abruption and chorioamnionitis. We also examined GA at time of IUFD, donor vs recipient IUFD, and latency times from FLA to IUFD or PPROM.

### Delivery and neonatal outcomes

Neonatal outcomes examined were number of live deliveries, complete twin deliveries, GA at delivery, birth weights, and incidences of neonatal mortality and morbidities. We also examined differences in GA at birth in cases of PPROM, neonatal mortality, morbidity and IVH compared to cases without these complications. Incidences of discrete morbidity categories and severity of IVH were also measured.

For all procedural, perinatal, delivery and neonatal outcomes, results between selective FLA and Solomon FLA groups were compared.

### Statistical analysis

Continuous variables were assessed for normality of distribution using Shapiro-Wilk test are presented as means with standard deviations (SD) or medians based on presence of normal distribution with interquartile ranges (IQR). Differences between continuous variables were tested using Wilcoxon-Mann Whitney test. Categorical variables were presented as count and percentages along with their 95% confidence intervals where applicable. Associations between categorical variables were tested using the Fisher’s exact *t* test or Chi-square test when appropriate. For all tests of significance, *p* ≤ 0.05 were considered statistically significant. Power analyses were performed for outcomes that were potentially clinically significant but not statistically significant. All analyses and graphic illustrations were conducted with Microsoft Excel (Excel 365; Microsoft Corp., Redmond, Washington, USA), GraphPad Prism 7 (GraphPad Software, San Diego, CA, USA) or SAS/STAT software, Version 9.4 of the SAS System for Windows (SAS Institute Inc, Cary, NC).

## Results

During the study period, 77 pregnant women received FLA for treatment of TTTS, of which 1 was excluded due to loss to follow up; thus, 76 maternal patients and their 128 liveborn neonates were included in this study (Fig. 1). A total of 40 maternal patients underwent selective FLA and 36 patients underwent Solomon FLA (Table 1). Due to transfer of care following immediate post-procedural monitoring, there was limited perinatal history, delivery, and neonatal data for some patients (Fig. 1).

**Table 1:** Maternal demographics.

	Overall (*N* = 76)	Selective (*N* = 40)	Solomon (*N* = 36)	*P* value^a^
Age (SD)	30.7 (6.1)	30.3 (6.8)	31.3 (5.3)	0.701
BMI (SD)	28.6 (6.8)	28.8 (8.2)	28.4 (4.9)	0.812
Medical History n (%):				
Pre-eclampsia	4 (5.3)	3 (7.5)	1 (2.8)	0.617
Gestational Diabetes	5 (6.6)	2 (5.0)	3 (8.3)	0.663
Cervical Insufficiency	4 (5.3)	3 (7.5)	1 (2.8)	0.617
Preterm Labor	4 (5.3)	0 (0.0)	4 (11.1)	*0.046
Drug Use	5 (6.6)	3 (7.5)	2 (5.6)	1.000
Mental Health	4 (5.3)	2 (5.0)	2 (5.6)	1.000
In Vitro Fertilization	3 (4.0)	1 (2.5)	2 (5.6)	0.601
Advanced Maternal Age	16 (21.1)	8 (20.0)	8 (22.2)	1.000
Chronic Health Condition	12 (15.8)	7 (17.5)	5 (13.9)	0.759
TTTS Staging n (%):				0.528
Stage 1	12 (15.7)	7 (17.5)	5 (13.9)	
Stage 2	17 (22.4)	6 (15.0)	11 (30.6)	
Stage 3	41 (53.9)	23 (57.5)	18 (50)	
Stage 4	5 (6.6)	4 (10.0)	1 (2.8)	
TAPS	1 (1.3)	0 (0.0)	1 (2.8)	

Maternal patients in the Solomon group had a higher incidence of history of preterm labor *p = 0.046.

^a^Statistical analyses between maternal patients undergoing selective vs Solomon techniques. Mean age and BMI were tested using Mann–Whitney *t* test, medical histories, and TTTS staging distribution were tested using categorical analyses.

### Maternal demographics

Characteristics of maternal patients are displayed in Table 1. Mean age of patients at time of procedure was 30.7 (SD 6.1) years with a mean BMI of 28.6 (SD 6.8). The most frequent medical histories were advanced maternal age and existing chronic health conditions. Chronic health conditions varied and included asthma (*n* = 3), chronic hypertension (*n* = 5), hypothyroidism (*n* = 4) with one individual with associated history of thyroid cancer, type 2 diabetes milletus (*n* = 2), anemia (*n* = 6), and history of herpes simplex virus (*n* = 2). Other significant medical conditions of individual patients were sickle cell anemia requiring intervention during pregnancy, invasive ductal carcinoma on chemotherapy, uterine fibroids, scleroderma, adenocarcinoma and polycystic ovarian syndrome, and congestive heart failure. The distribution of TTTS staging at time of the procedure is shown in Table 1: 12 (15.7%) stage 1, 17 (22.4%) stage 2, 41 (53.9%) stage 3, 5 patients (6.6%) stage 4, and 1 (1.3%) TAPS. There were no significant differences in characteristics of maternal patients between the selective and Solomon FLA groups, except that the Solomon FLA group had a statistically higher incident of prior history of preterm labor with 4 (of 36) patients compared to 0 (of 40) patients in the selective FLA group (*p* = 0.046).

### Procedural and perinatal data

Procedural data and perinatal outcomes are shown in Table 2. The median GA at time of FLA was 20.8 weeks (IQR 18.1–22.9); there were no differences between selective and Solomon FLA groups (*p* = 0.263). The incidences of post laser TAPS and TTTS recurrence were each 4/76 cases (5.3%), and were predominantly in the selective FLA group. However, the difference between selective and Solomon FLA groups, 3 (7.5%) TAPS with 4 (10%) TTTS recurrence and 1 (2.8%) TAPS and 0 (0%) TTTS recurrence respectively, were not statistically significant (*p* = 0.617 and 0.117, respectively).

**Table 2:** Perinatal Outcomes.

	Overall (*N* = 76)	Selective (*N* = 40)	Solomon (*N* = 36)	*P* value^a^
GA at time of FLA (IQR)	20.8 (18.1–22.9)	21.9 (18.3-23.0)	20.0 (18.0-21.9)	0.263
Post Laser TAPS n (%)	4 (5.3)	3 (7.5)	1 (2.8)	0.617
TTTS recurrence n (%)	4 (5.3)	4 (10.0)	0 (0.0)	0.117
IUFD n (%)	20 (26.3)	11 (27.5)	9 (25.0)	1.000
Donor n (%)	13 (17.1)	7 (17.5)	6 (16.7)	
Recipient n (%)	4 (5.3)	2 (5.0)	2 (5.6)	
Both n (%)	3 (3.9)	2 (5.0)	1 (2.8)	
Median GA at time of IUFD (IQR)	20.7 (18.6-25.0)	20 (17.9-23.0)	22.6 (20.3-25.7)	0.168
Median latency in days from FLA to IUFD (IQR)	1 (0.3-23.3)	1 (0.0–9.0)	13.5 (0.5–46.5)	0.399
PPROM n (%)	22 (31.4)	11 (28.9)	11 (34.4)	0.805
Median GA at time of PPROM (IQR)	27.6 (24.9-32.3)	25.3 (24.6-29.6)	32.3 (24.9-33.7)	0.109
Median latency in weeks from FLA to PPROM (IQR)	6.94 (2.8–10.5)	4.1 (1.9–6.7)	10.1 (7.2-12.7)	*0.017
Chorioamnionitis n (%)	6 (8.6)	2 (5.3)	4 (12.5)	0.326
Placental abruption n (%)	6 (8.6)	3 (7.9)	3 (9.4)	0.870
Available data^b^ n	70	38	32	

Latency time from FLA to PPROM was significantly longer in the Solomon group compared to the selective group *p = 0.017.

^a^Statistical analyses between maternal patients undergoing selective vs Solomon techniques. Gestational ages at time of respective events and comparison of time latencies from FLA to IUFD and PPROM were examined using Mann–Whitney *t* tests. Incidence of post laser TAPs, TTTS recurrence, IUFD, PPROM, chorioamnionitis, and placental abruption were analyzed by categorial analyses.

^b^Available data in (n) cases for perinatal complications: PPROM, chorioamnionitis, and placental abruption.

The incidence of IUFD was 20 (26.3%) and were predominantly single fetal deaths 17 (22.4% of FLA and 85% of IUFD cases), most of which were donor fetus 13 (17.1% of FLA and 65% of IUFD cases) (Table 2). There were 3 cases (3.9%) of FLA where both fetuses were lost. The median GA at time of fetal loss was 20.7 weeks (IQR 18.6-25.0) with most fetal losses occurring shortly after the procedure as median latency time from FLA to IUFD was 1 day (IQR 0.3-23.3). Only 7 (9.2% of FLA, 35% of IUFD) cases were pregnancies also complicated by PPROM, but events were not concurrent with IUFD, and all PPROM events occurred at later time points. As shown in Table 2, there were no differences in incidence of IUFD between the selective and Solomon FLA groups (*p* = 1.000). While median latency times suggest a possible difference between selective and Solomon FLA groups, 1 day (IQR 0.0–9.0) and 13.5 days (IQR 0.5–46.5), respectively, this difference was not significant (*p* = 0.399); though, of note, this outcome also had low power (0.325).

In regard to perinatal complications, the incidence of PPROM was 22 (31.4%) of cases with a median GA at time of PPROM of 27.6 weeks (IQR 24.9–32.3); there was no difference between the selective and Solomon FLA groups (Table 2). The median latency time from FLA to PPROM was 6.94 weeks (IQR 2.8–10.5). Interestingly, the selective FLA group had a significantly shorter latency time 4.1 weeks (IQR 1.9–6.7) compared to the Solomon FLA group 10.1 weeks (IQR 7.2-12.7) (*p* = 0.017). The incidences of chorioamnionitis and placental abruption were low, both 6 (8.6%) cases, with no difference between the procedural groups (*p* = 0.33 and *p* = 0.87, respectively).

### Delivery and neonatal data

**Fig. 2:**
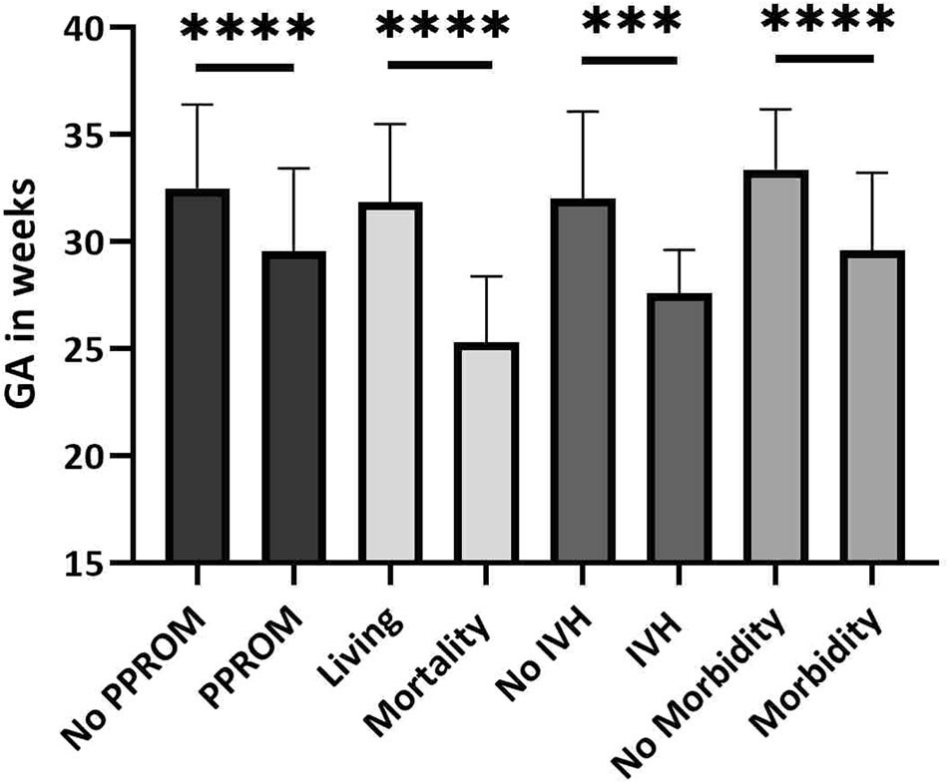
Gestational Ages at Birth for PPROM and neonatal complications. Pregnancies complicated by PPROM had statistically significant lower GA at time of birth ****p < 0.0001. Cases of neonatal mortality, IVH, and overall morbidities were also associated with significantly lower GA at time of birth ***p = 0.001 ****p < 0.0001. Error bars indicate SD. Statistical analyses by Mann–Whitney *t* tests.

Delivery data and neonatal outcomes are shown in Table 3. There were 73 (96%) [95% CI: 88.9–99.2%] liveborn deliveries of at least 1 neonate, 56 (73.7%) [95% CI: 62.3–83.1%] were full twin sets and there was a total of 129 liveborn neonates (67 in the selective FLA group and 62 in the Solomon FLA group). Most maternal patients delivered by cesarean section 49 (64.5%); there were no differences between the procedural groups (*p* = 0.63). The median GA at birth was 33.1 weeks (IQR 28.0–35.0) with a median birth weight (BW) of 1.52 kg (IQR 0.94–2.01). The median GA at birth was significantly lower in cases of PPROM compared to cases without PPROM, 30.4 weeks (IQR 25.4–33.3) versus 34.4 weeks (IQR 28.3–35.1), respectively (*p* < 0.0001) (Fig. 2). The median GA was significantly lower in the selective FLA group compared to the Solomon FLA group, 31.2 weeks (IQR 27.0–35.0) with BW 1.27 kg (IQR 0.76–1.87) and 34.0 weeks (IQR 30.3–35.1) with BW 1.75 kg (IQR 1.22–2.18), respectively (*p* = 0.029 and *p* = 0.005 for GA and BW, respectively). The incidence of twin discordance, wherein there was a >20% difference in BW, was 17 (37.8%) with no difference between the selective and Solomon FLA groups (*p* = 1.000).

**Table 3:** Neonatal outcomes.

	Overall (*N* = 76)	Selective (*N* = 40)	Solomon (*N* = 36)	*P* value^a^
Live deliveries n (%)	73 (96.0)	39 (97.5)	35 (97.2)	0.925
Twin sets n (%)	56 (73.7)	29 (72.5)	27 (75.0)	1.000
Total liveborn neonates n	129	67	62	1.000
Vaginal n (%)	18 (23.7)	8 (20.0)	9 (25.0)	0.751
Cesarean section n (%)	49 (64.5)	28 (70.0)	22 (61.1)	0.630
Delivery mode unknown n	9 (11.8)	4 (10.0)	5 (13.9)	
Median GA at birth (IQR)	33.1 (28.0–35.0)	31.2 (27.0–35.0)	34.0 (30.3–35.1)	*0.029
Median Birth weight kg (IQR)	1.52 (0.94–2.01)	1.27 (0.76–1.87)	1.75 (1.22–2.18)	**0.005
Twin discordance^b^ n (%)	17 (37.8)	10 (40.0)	8 (38.0)	0.925
BW twin sets available data n	45	25	21	
Neonatal mortality n (%)	12 (9.3)	7 (10.4)	5 (8.1)	0.756
Median CGA at time of death (IQR)	26.4 (22.1–31.6)	25.7 (22.4–29.1)	31.6 (19.3–42.0)	0.900
Donor n (%)	5 (3.9)	4 (6.0)	1 (1.6)	
Recipient n (%)	5 (3.9)	1 (1.5)	4 (6.5)	
Donor/Recipient unknown n (%)	2 (1.6)	2 (3.0)	0 (0.0)	
Neonatal morbidities^c^ n (%)	43 (48.3)	31 (75.6)	12 (40.0)	**0.0065
Sepsis n (%)	12 (13.5)	10 (24.4)	2 (6.7)	*0.028
Early Onset n (%)	3 (3.4)	3 (7.3)	0 (0.0)	0.245
Late Onset n (%)	9 (10.1)	7 (17.1)	2 (6.7)	0.166
Cardiac Dysfunction n (%)	16 (18.0)	10 (24.4)	6 (20.0)	*0.020
Renal Dysfunction n (%)	14 (15.7)	10 (24.4)	4 (13.3)	0.158
Morbidity available data n	89	41	30	
Polycythemia n (%)	11 (13.8)	7 (15.6)	4 (11.4)	0.533
Recipient status n (%)	7 (8.8)	4 (8.9)	3 (8.6)	
Anemia n (%)	11 (13.8)	8 (17.6)	3 (27.3)	0.208
Donor status n (%)	6 (7.5)	5 (11.1)	1 (2.9)	
Blood transfusion n (%)	7 (8.8)	5 (11.1)	2 (5.7)	
Hematology available data^d^ n	80	45	35	
IVH^e^ n (%)	13 (18.3)	10 (24.4)	3 (10.0)	0.078
IVH Available data n	71	41	30	
Grade I unilateral	5 (7.0)	3 (7.3)	0 (0.0)	
Grade II unilateral	1 (1.4)	1 (2.4)	2 (6.7)	
Grade IV unilateral	1 (1.4)	1 (2.4)	0 (0.0)	
Grade I bilateral	2 (2.8)	2 (4.9)	0 (0.0)	
Grade III bilateral	1 (1.4)	1 (2.4)	0 (0.0)	
Grade IV bilateral	1 (1.4)	0 (0.0)	1 (3.3)	
Bilateral, grades variable	2 (2.8)	2 (4.9)	0 (0.0)	
Periventricular leukomalacia	4 (5.6)	3 (7.3)	1 (3.3)	

GA at birth significantly higher in the Solomon group *p = 0.029. Birth weight of neonates significantly higher in the Solomon group **p = 0.005. There were more individual neonates with morbidities in the selective group compared to the Solomon group **p = 0.0065. In a breakdown of the morbidities studied, only the frequency of sepsis and cardiac dysfunction, which was higher in the selective group, reached statistical significance *p = 0.020.

^a^Statistical analyses between selective FLA and Solomon FLA groups. GA at birth and birth weights were examined using Mann–Whitney *t* tests. Incidence of events (i.e. delivery events, neonatal mortality and morbidities) were analyzed by categorical analyses.

^b^Twin discordance defined as a weight discrepancy of >20%. Percentage reflected of available neonate weight data of twin sets.

^c^Number of individual neonates with single or multiple morbidities. Percentage reflected of available data *n* = 89.

^d^Not all neonates have hematological labs obtained upon delivery.

^e^IVH results based on available data, wherein head ultrasound data was known (71). While there were more cases of IVH in the selective group compared to the Solomon group, this difference did not reach significance *p* = 0.078.

The incidence of neonatal mortality was 12 (9.3%) of 129 neonates with a median CGA at time of death of 26.4 weeks (IQR 22.4-31.6) (Table 3). There was no difference between the procedural groups (*p* = 0.765). The breakdown of TTTS status of neonates were evenly split, 5 each for donor and recipient statuses with 2 statuses unknown. Only one neonatal death was associated with a postoperative complication (post laser TAPS). The median GA at birth for cases of neonatal mortality were significantly lower than for neonates who survived, 25.1 weeks (IQR 23.0-27.7) versus 32.9 weeks (IQR 28.3–35.0), respectively (*p* < 0.0001) (Fig. 2). Three neonates were previable at time of delivery: 22.4 weeks twins (institutional policy was resuscitation ≥23 weeks at this period) and 19.3 weeks singleton and died shortly after birth. One neonate developed severe respiratory failure shortly after birth and the family elected for comfort care after aggressive intervention. A number of neonates died from complications associated with prematurity: one neonate had necrotizing enterocolitis totalis; two neonates had severe pulmonary hypoplasia or bronchopulmonary dysplasia with clinical deterioration; and two neonates had bilateral IVH, one of whom also had hydrops. The oldest neonate passed at 70.6 weeks CGA from sudden onset ventricular tachycardia that did not recover despite cardiorespiratory resuscitation efforts.

The median GA at birth for neonates with morbidities was significantly lower than neonates with unremarkable clinical courses, 28.3 weeks (IQR 26.9-33.7) versus 34.6 weeks (IQR 31.3–35.1), respectively (*p* < 0.0001) (Fig. 2). As shown in Table 3, the overall incidence of neonatal morbidities of individual with at least one morbidity was 43 (48.3%) and was significantly higher in the selective FLA group compared to the Solomon FLA group, 31 (75.6%) versus 12 (40%) individual neonates, respectively (*p* = 0.0065). In terms of specific morbidities, overall incidence of sepsis was 12 (13.5%) with more cases in the selective FLA group with 10 (2.4%) cases than the Solomon FLA group with 2 (6.7%) cases (*p* = 0.028). Incidence of at least one parameter of cardiac dysfunction was 16 (18%) cases, which was also higher in the selective FLA group compared to the Solomon FLA group, 10 (24.4%) versus 6 (20%), respectively (*p* = 0.02). Individual neonates were usually affected by more than one type of cardiac dysfunction as defined in the methods. The most common types of cardiac dysfunction were clinically significant PDA (13, 14.6%), hypotension (11, 12.5%) and presence of congenital heart disease (10, 11.2%), which included 2 (2.2%) cases of ventricular septum defect (VSD) and 6 (6.7%) cases of pulmonary valve stenosis, many requiring therapeutic valvuloplasty. There were 3 (3.4%) cases of arrythmias and 4 (4.5%) cases of persistent pulmonary hypertension, of which 3 were the donor twin. Other types of cardiac dysfunction were 1 case of endocarditis, 2 cases of hydrops, and 1 case with history of fetal heart failure with recovery of heart function in the neonatal period. The incidence of IVH was 13 (18.3%). Though neonates in the selective FLA group had a higher incidence of IVH of 10 (24.4%) cases compared to neonates in the Solomon FLA group with 3 (10%) cases, this difference was not statistically significant (*p* = 0.122); but this outcome also had low power (0.1823) (Table 3). The average GA at birth for neonates with IVH was also compared to neonates without IVH and was shown to be statistically significant lower with median GA of 27.7 weeks (IQR 25.7–28.8) versus 33.9 weeks (IQR 28.6-35.1) respectively (*p* = 0.0001) (Fig. 2). As shown in Table 3, the incidence of other morbidities was 14 (15.7%) for renal dysfunction, and 11 (13.8%) for both polycythemia and anemia immediately after delivery with no differences between the procedural groups (*p* = 0.158, *p* = 0.208, *p* = 0.533 for renal dysfunction, anemia and polycythemia, respectively). Congenital renal anomalies were present in 6 (6.7%) neonates and 12 (13.5%) were affected by AKI and/or renal failure. Expectantly, most cases of polycythemia at birth were in former recipient fetuses (7 cases, 8.8% overall and 63.6% of polycythemia cases), and most cases of anemia at birth were in former donor fetuses (6 cases, 7.5% overall and 54.5% of anemia cases) (Table 3). 7 neonates (8.8% overall and 63.6% of anemia cases) required blood transfusions.

## Discussion

FLA is the preferred treatment for advanced stages of TTTS and most interventions occur between 16- and 26-weeks of gestation [1, 4, 7, 9, 10, 12–14, 19]. Cases presenting after 26 weeks are less common and more technically challenging due to increased amniotic fluid turbidity and larger vessel diameter [1]. However, the procedure is feasible and has been successfully performed at our and other institutions with outcomes comparable to interventions at earlier GA [1, 20]. Many cases of stage 1 TTTS remain stable or even regress, but may advance to more severe staging necessitating intervention [1, 15]. Previous studies showed that up to 60% of conservatively managed stage 1 TTTS cases progressed and only FLA was protective against fetal loss or very preterm delivery (<26 weeks) [15, 21]. TAPs though more commonly a post laser procedural complication can occur spontaneously in up to 5% of monochorionic twins due to chronic transfusion from one twin to the other resulting in anemia and polycythemia without oligohydramnios or polyhydramnios [1]. Patients with TAPS, like stage 1 TTTS, can be managed conservatively but surgical FLA is also an option [22]. When deciding whether or not to surgically intervene in stage 1 TTTS or TAPS, providers must consider the overall health of and presence of symptomatology in pregnant women, as well as patient availability and accessibility for close follow up, which may be an issue in large referral centers like our institution wherein patients may travel great distances for consultations and routine close monitoring may not be practical or financially feasible.

Overall, our rates of procedural complications, post laser TAPS and recurrent TTTS, were low (both 4%) and comparable to those reported in previous literature [8, 13, 14, 19]. Previous studies showed lower occurrences in post laser TAPS and TTTS recurrence in patients who underwent Solomon FLA compared to those who underwent Selective FLA [8, 13, 14, 19]. There was no statistical difference between procedural groups in our study, likely due to low numbers overall. Findings shown in previous studies are likely due to the larger proportion of persisting twin-twin anastomoses in selective FLA, as only connections visualized are eliminated, and deeper or smaller vascular connections are often missed [12, 15].

Overall, 26.3% of cases experienced IUFD of at least one twin, majority of which were the donor fetus. Higher death rate of donors, and more frequent loss of a single twin as opposed to double twin loss are congruent with existing literature [9, 11, 19]. Similar to our study, the majority of IUFD cases occurred within a few days of FLA, particularly for selective FLA cases [12, 19]. Though the differences between procedural groups did not reach significance in our study, our statistical outcomes may be in part, due to low power. Dual losses are comparatively less common following FLA, occurring at a rate of 6% in experienced centers; this is congruent with our rate of dual loss (3.9%) [9, 11, 19]. Interestingly, Stirnemann et al. (2018) previously showed that fetal loss was strongly associated with PPROM <20 weeks. While some of our cases (35%) of IUFD were associated with pregnancies complicated by PPROM, these events did not occur concurrently and all patients developed PPROM at a later GA [9].

The most common perinatal complication associated with TTTS and FLA is PPROM [1, 2, 9]. The postoperative perinatal complications are largely related to issues of membrane integrity and placental tissue health and injury following FLA [1, 15]. Previous studies showed the risk of PPROM increased over time from surgery to delivery and occurs in up to 39% of cases by 34 weeks, which is congruent with our incidence of 31.4% [1, 2, 9]. Similar to our results, the original RCT studying selective and Solomon techniques did not show differences in rates of PPROM [14]. However, Solomon technique has been associated with longer interval time between FLA and PPROM, which is congruent with our results [12]. Given that early PPROM (<20 weeks) is associated with high rates of fetal loss and very early prematurity, this suggests better perinatal outcomes for Solomon FLA despite comparable PPROM rates [9].

Other perinatal complications studied occurred less frequently; 8.6% for both chorioamnionitis and placental abruption. Furthermore, we did not find significant differences between procedural groups despite higher incidences of placental abruption for Solomon FLA reported in the literature [15, 17, 19]. This reason of this difference in rates of placental abruption is unclear, but may be related to specific procedural parameters, such as surgical time and laser energy strength, which are not examined in this study.

Encouragingly, our survival rates were high, 96% [95% CI: 88.9 – 99.2%] of at least 1 twin and 73.7% [95% CI: 62.3–83.1%] of twin sets; outcomes which are comparable to other high-volume centers that similarly see survival of at least 1 twin of >90% and double twin survival of >70% [7, 9, 10, 13, 19, 20]. Previous studies reported that the Solomon technique showed improved neonatal survival [12, 13]. Stirnemann etal (2018) has shown that high volume centers have seen increases of survival to delivery overtime despite the frequency of PPROM, which may possibly be associated with the use of Solomon technique as opposed to selective FLA [9]. However, it is important consider the impact of a learning curve effect of experienced providers at high volume centers, which likely contributes to improved survival outcomes overtime and may diminish the possible relative superiority of survival rates of the Solomon technique over survival rates the selective technique [23]. Indeed, there was no difference in survival rates between procedural groups in our study as rates were similarly high for both.

Our median GA at birth was 31.6 weeks, which is within the average range of 32-34 weeks in previous reports [7, 10, 13, 15, 17]. Notably, the latency following PPROM, median GA and birth weights were higher in the Solomon FLA group, which supports the superiority of outcomes with the Solomon technique. This finding is clinically meaningful despite the higher incidence of preterm labor and PPROM which may be related to the increase in laser energy and resultant placenta tissue injury with Solomon FLA.

However, this difference in GA at birth has not been shown in previous studies [14, 17, 19]. Although, Sago et al. (2010) showed that following Solomon FLA, the recipients had comparatively lower birth weights, which was likely reflectively of resolution of volume overload and over-circulation with more complete elimination of communicating anastomoses [19].

Our incidence of neonatal mortality (9.4%) was similar to previous reports of about 10%, and frequently related to extreme prematurity [11, 24]. Tollenaar et al. (2020) found higher mortality rates in donors, specifically in post-laser donor neonates (15% vs 6% in recipients) [11]. In our study, only 1 neonatal death was associated with post laser TAPS and incidences of death between former donors and recipients were the same. Literature shows variation in neonatal mortality outcomes when comparing selective and Solomon FLA. Some literature report, like in our study and despite higher GA at birth and a higher birth weight in Solomon cases, saw no difference in mortality rates between procedures [11, 15]. However, Sago et al. (2010) found lower mortality rates in Solomon FLA cases [19].

The neonatal morbidities studied were chosen based on previously identified associations TTTS and FLA [1–4, 11, 15, 24]. Our incidence of individual neonates with at least one morbidity was 48.3% and associated with significantly lower GA at birth; this is congruent with literature reports that many neonatal morbidities in TTTS and FLA are strongly associated with lower GA and in conjunction, lower birth weights [2, 11, 24]. Given higher GA and birth weight in our Solomon FLA group, expectantly, incidence of neonatal morbidities was statistically significantly lower. A higher incidence of sepsis was seen in the selective FLA group, most of which, were late onset sepsis, and thus, may be related to increased vulnerability to infection in a younger GA cohort. Cardiac dysfunction of fetuses with TTTS is related to either prematurity or due to differences in volume loading and hormonal aberrations in fetal and placental tissues and usually improve after FLA [1, 11]. Common findings are dysfunction associated with prematurity such as PDA and clinically significant hypotension, CHD with pulmonary valve stenosis or atresia, persistent pulmonary hypertension, or hydrops [1, 2, 15]. Our incidences of cardiac dysfunction are similar; most of our cases of CHD were also pulmonary valve stenosis, many of whom required therapeutic valvuloplasty in the neonatal period.

Cerebral injury (CI) is of particular interest as rate of CI are high in TTTS, with highest risk for single surviving twins after in utero demise of their co-twin, occurring up to 27% [2, 4]. This is due to severe anemia and hypovolemia caused by acute transfer of blood from the surviving fetus to the dead or dying sibling via existing vascular anastomoses [2, 4]. FLA is protective against this and lowers the incidence of CI though may still occur at a rate of 2-18%, which is reflected in our study with an incidence of IVH or 18.3% [2, 4]. Donor and recipient twins are affected equally and no differences have been seen between selective and Solomon FLA techniques [2, 11]. Although our study has more cases of IVH in the selective FLA group in than the Solomon FLA group, the difference was not statistically significant, which may have been due to insufficient power. The presence of CHD has been associated with CI, but the most important factors have been shown to be prematurity and low birth weight [2]. This is congruent with our study as neonates with IVH had lower median GA at birth.

Given that renal dysfunction of often seen in higher stages of TTTS, we found congenital renal anomalies present in 6.7% of neonates and 13.5% of neonates were affected by AKI or renal failure [2]. Our incidences of hematologic pathology were 13.8% for both polycythemia and anemia with no differences between the procedural groups. Most cases of polycythemia were in former recipient fetuses and most cases of anemia were in former donor fetuses, which suggests the significant presence of persistent vascular connections in pregnancies that did not meet formal criteria for diagnosis of post laser TAPS. 63.6% of anemic neonates required blood transfusions immediately following birth, similar to rates reported previously [11].

Our study carries the known limitations of retrospective studies, and was conducted in a single center, thus potentially limiting is generalizability. Data was limited for a number of maternal patients and their neonates, who had obstetrical and newborn care elsewhere following the immediate postoperative period. Due to sample size limitations, this study may not have been sufficiently powered to adequately examine certain parameters, such as for rates of IVH. Considerable literature has also been dedicated to examining longer term outcomes, particularly with regards to neurodevelopmental impairments, which was not included in the scope of the study, but warrants future exploration given the strong associations between prematurity and cerebral injury [1, 4, 15, 24].

In summary, this study demonstrated strong perinatal and neonatal outcomes for the treatment of TTTS and FLA for our single center institution; we did not detect differences in some outcomes compared to many high-volume centers but this may be due to small sample size which could result in clinically significant differences remaining undetected. Conventionally, the Solomon technique has been considered the superior technique due to lower procedural complication and improved survival rates despite possible worse placental tissue damage [9, 12–14]. However, our study suggests that in high volume centers with experienced providers, this difference may diminish. Though, despite similar overall survival rates, our results still support the superiority of the Solomon technique in other respects: longer interval time between FLA and PPROM when it occurs, higher GA at birth with higher birth weights, and consequently, lower rates of neonatal morbidities. Future directions include more obstetrical focus, examining outcomes in relation to specific procedural parameters such as duration of surgical procedure, strength of laser energy, amnioreductions and the role of learning curve effects. It would also be important to evaluate long term outcomes for our neonatal population, which particular focus on neurodevelopmental outcomes.

## Data Availability

The datasets generated during and/or analyzed during the current study are available from the corresponding author on reasonable request.
